# The protective effect of inactivated *Flavobacterium columnare* vaccine in grass carp (*Ctenopharyngodon idellus*)

**DOI:** 10.3389/fimmu.2023.1162975

**Published:** 2023-07-13

**Authors:** Wenjie Guo, Rui Han, Weizhen Xu, Zijun Lu, Yanwei Li, Xueming Dan, Zequan Mo

**Affiliations:** ^1^ University Joint Laboratory of Guangdong Province, Hong Kong and Macao Region on Marine Bioresource Conservation and Exploitation, College of Marine Sciences, South China Agricultural University, Guangzhou, China; ^2^ Henry Fok School of Biology and Agriculture, Shaoguan University, Shaoguan, China

**Keywords:** grass carp, *Flavobacterium columnare*, vaccine, adjuvant, relative percent survival

## Abstract

*Flavobacterium columnare*, which causes columnaris disease, is responsible for significant mortality in grass carp. Vaccination is a safe and effective measure to combat this disease, and this study aimed to investigate the immune protective effects of different treatments using an inactivated *F. columnare* vaccine. The vaccine was prepared by inactivating the bacteria with 0.05% formaldehyde at 4°C for 24 hours. The experiments involving grass carp were divided into two parts. In Experiment 1, the immune effects of two isolates, JX-01 (genomovar I) and MU-04 (genomovar II), were compared, along with the impact of white oil adjuvant and the number of immunizations. The results showed that when the white oil adjuvant was used as a booster, the relative percent survival (RPS) of the JW2 group and MW2 group after 8 weeks of the first immunization was 34% and 61%, respectively. In Experiment 2, only the MU-04 (genomovar II) isolate was used as an antigen, with the white oil adjuvant as a booster. The effects of different doses (CFU=10^8^, 10^7^, and 10^6^ bacteria/mL) on immune responses were compared, and the RPS values in the MW6, MW7, and MW8 groups after 4 weeks of the first immunization were found to be 38%, 57%, and 71%, respectively. Furthermore, in the cross-antigen protection experiment, the MW2 group exhibited an RPS of 55% against the JX-01 isolate, which was significantly higher than the control group (33%). These findings suggest that an inactivated vaccine comprising an appropriate antigen isolate when administered with a white oil adjuvant as a booster, can provide effective protection in grass carp.

## Introduction

*Flavobacterium columnare* is a Gram-negative bacterium responsible for causing columnaris disease in freshwater fish worldwide (1). Various fish species are susceptible to this bacterium, and infection often manifests as gill necrosis, skin lesions, and fin rot (2). Vaccination has emerged as a crucial approach to prevent and manage this disease.

Early vaccination strategies involved the use of formalin-killed bacterins, both with and without adjuvants, but their efficacy was limited (3). In the United States, a commercial live-attenuated vaccine has been developed to protect against columnaris in catfish (4). This vaccine has shown promise in preventing columnaris disease in the fry of channel catfish (*Ictalurus punctatus*) and largemouth bass (*Micropterus salmoides*) (5). However, live vaccines carry the risk of reversion. To overcome this, researchers have explored the generation of *F. columnare* ghosts using the lysis gene E, which results in empty cell envelopes that offer immune protection. However, achieving a 100% lysis rate of the bacterium ghosts (BGs) strain in a short time remains challenging and may potentially lead to the release of live *F. columnare* (6). For a commercial vaccine to be widely employed, it must meet the criteria of safety, affordability, and effectiveness.

The grass carp (*Ctenopharyngodon idellus*) is a commercially important species in Chinese aquaculture, contributing to approximately 10.5% of global fish production (7). However, the outbreak of columnaris disease poses a significant threat to grass carp farming (8). The current reliance on antibiotics for controlling columnaris carries the risk of drug-resistance development and environmental pollution. Therefore, vaccination has emerged as an alternative method for columnaris control.

Previous studies have identified three genomovars of *F. columnare* in grass carp using restriction fragment length polymorphism of the 16S rDNA gene (16S-RFLP). From grass carp affected by columnaris in China, we obtained 45 bacterial isolates, with the majority belonging to genomovar I, and only a few belonging to genomovar II and III. Phylogenetic analysis confirmed the genetic differences between the three genomovar isolates and revealed a closer relationship between genomovar I and II (9). However, it remains unclear whether antigens from genomovar I and II can provide cross-protection.

The addition of an adjuvant to the vaccine can significantly enhance its immune protection. White oil emulsion is commonly used as a vaccine adjuvant in farm animals. It is known to form an oil-water structure that allows for the sustained release of vaccine antigens and consistent stimulation of the immune response, leading to higher antibody titers compared to naked antigens (10). Although some research has analyzed the effectiveness of the *F. columnare* vaccine, most of these studies were conducted on a laboratory scale (11). In the present study, we aimed to identify a more suitable formula for the practical production of an inactivated *F. columnare* vaccine by investigating its effectiveness in an outdoor pond setting. We tested two genomovars, MU-04 and JX-01, at various antigen concentrations. Additionally, we evaluated the supplementation of white oil adjuvant to develop an effective inactivated vaccine formula for grass carp against *F. columnare* infection.

## Materials and methods

### Bacterial strains and growth conditions

Previously isolated and characterized *F. columnare* isolates (9), namely, JX-01 (genomovar I) and MU-04 (genomovar II), were employed in this study. Methods of bacterial isolation: The inoculation ring was inserted into the internal tissue of grass carp and dipped in the isolates. Lines were drawn on the Shieh plate, and then the plate was incubated at a constant temperature of 28°C for 48 h. Due to the special morphology of the colonies of *F. columnare*, whether similar colonies were generated on the plate could be observed by the naked eye before preliminary determination, and the specific primers of *F. columnare* were used to further verify the bacteria by PCR. These two strains demonstrated a high infective capacity in grass carp (see **Supplementary Figure 1**), making them suitable candidates for the vaccine. The strains were preserved as glycerol stocks at -80°C and cultured on modified Shieh (MS) agar or broth with shaking at 200 rpm at 28°C for 20 hours (12). To retrieve *F. columnare* from the frozen glycerol, streaking onto Shieh medium was performed. After 48 hours of growth at 28°C, a single colony was inoculated into 60 mL of Shieh broth and incubated for 20 hours at 28°C. At various time points, aliquots of the cell culture were collected, and the cell density was measured at OD450. Plate counts of each strain (in triplicates) were conducted to determine the average number of colony-forming units per milliliter (CFU/mL), which were subsequently utilized for fish vaccination or challenge experiments.

### Fish

Healthy grass carp weighing an average of 15 g (n = 5000 fish) were acclimatized for one week and then distributed into cages measuring 2 m × 2 m × 2 m and placed in a pond. The water temperature was monitored daily and allowed to naturally fluctuate according to local climate conditions. Throughout the study, the fish were fed a commercial pelleted diet at a rate of 2% of their body weight per day. The fish were given 7 days to acclimate to the cage environment. Prior to conducting the infection and vaccination experiments, 20 fish were randomly selected, and bacterial isolates were obtained from their blood, liver, and spleen. The presence of *F. columnare* was determined by streaking the samples on modified Shieh agar. No *F. columnare* was detected in any of the examined fish.

### Expression of recombinant grass carp IgM and antibody development

A codon-optimized grass carp IgM heavy chain sequence (DQ417927.1) for *Escherichia coli* expression was synthesized by Generay Biotech Co., Ltd. (Shanghai) and cloned into the pET32a-ΔTRX expression vector (prepared in our lab), resulting in the generation of pET32a-ΔTRX-grass carp-IgM (see **Supplementary Figure 2**). The pET32a-ΔTRX-grass carp-IgM plasmid was isolated and transformed into E. coli BL21(DE3) cells. Positive bacterial cells were induced with 1 mM isopropyl β-D-1-thiogalactopyranoside (IPTG). The grass carp recombinant IgM protein (Grass carp rIgM) was purified using a nickel nitrilotriacetic acid column (Ni-NTA; Qiagen, Germany).

The purified Grass carp rIgM protein was emulsified with Freund’s complete adjuvant (FCA), and 1 mg of the protein was injected into New Zealand white rabbits weighing approximately 1.3 kg. The rabbits were then boosted with 0.5 mg of Grass carp rIgM in Freund’s incomplete adjuvant (FIA) on two separate occasions. Serum was prepared, and the polyclonal antibody (pAb) titers were determined using enzyme-linked immunosorbent assays (ELISAs) with Grass carp rIgM as the antigen. Rabbit immunoglobulin G (IgG) was purified from rabbit antiserum using protein A agarose (Beyotime, Haimen, Jiangsu, China) according to the manufacturer’s instructions.

### Western blot analysis

Natural grass carp serum samples (diluted 1:20) were electrophoresed using a 10% SDS-PAGE gel and transferred to a polyvinylidene fluoride (PVDF) membrane. The PVDF membranes were blocked with 5% skim milk for 1 hour at 37°C. For the detection of grass carp IgM binding antibodies, the membranes were incubated with rabbit anti-grass carp rIgM antibody (1 mg/ml), followed by detection with HRP-conjugated anti-rabbit IgG (1 mg/ml, CST). The resulting bands were visualized using the SuperSignal West Pico Chemiluminescent Substrate.

### Vaccine preparation and immunization

Bacterial cultures used for vaccine preparation were grown in Shieh broth at 28°C for 20 hours. To determine the optimal inactivation conditions, bacteria were exposed to 0.025%, 0.05%, and 0.1% formaldehyde and incubated at 4°C, 28°C, and 37°C for 24 or 48 hours. Three setups were prepared in parallel for each group. After inactivation, 100 μL of the culture was used to coat plates with Shieh medium. Lack of colony growth after 48 hours at 28°C was considered an indicator of a safe vaccine (13). Twenty grass carp were randomly selected for intraperitoneal injection of 300 μL of the inactivated vaccine and observed for any abnormalities over the course of one week.

Immunogens were emulsified by mixing a white oil adjuvant (composed of 93% white oil, 2% tween-80, and 5% Span-80) with bacterial culture at a ratio of 2:1 (10). Each fish received an intraperitoneal (IP) injection of 0.1 mL of the vaccine to evaluate its protective effect. Booster immunization was administered two weeks after the initial immunization using the same vaccine and dose as the initial immunization. This study is divided into two parts: **Table 1** presents the effect of different genomovars, the addition of the white oil adjuvant, and the efficacy of booster immunization, while **Table 2** presents the effect of different bacterial concentrations on immunity.

**Table 1:** Experiment 1: To study the effect of different immune procedure on immunity.

Group	Strain^a^	Adjuvant	Dose number	Vaccination dose (CFU/mL)	Number
JW1	JX-01	White oil	Single	1.7 × 10^8^	200
JW2	JX-01	White oil	Booster^b^	1.7 × 10^8^	200
JP1	JX-01	PBS^c^	Single	1.7 × 10^8^	200
JP2	JX-01	PBS	Booster	1.7 × 10^8^	200
MW1	MU-04	White oil	Single	1.7 × 10^8^	200
MW2	MU-04	White oil	Booster	1.7 × 10^8^	200
MP1	MU-04	PBS	Single	1.7 × 10^8^	200
MP2	MU-04	PBS	Booster	1.7 × 10^8^	200
Control	/	PBS	Single	/	400

a F. columnare isolates: JX-01 (genomovar I) and MU-04 (genomovar II).

b The booster immunization was administered two weeks after the primary immunization.

c Immunogens were emulsified by mixing PBS with bacterial culture in a ratio of 2:1.

**Table 2:** Experiment 2: To study the effect of different bacterial concentrations on immunity.

Group	Strain	Adjuvant	Dose number	Vaccination dose (CFU/mL)	Number
MW6	MU-04	White oil	Booster	1.7 × 10^6^	200
MW7	MU-04	White oil	Booster	1.7 × 10^7^	200
MW8	MU-04	White oil	Booster	1.7 × 10^8^	200
WO	/	White oil	Booster	/	200
Control	/	PBS	Single	/	200

### Challenge of pathogen

Two weeks after the primary immunization, 30 grass carp were randomly selected from each cage and intraperitoneally (IP) injected with 100 μL of freshly cultured *F. columnare* in Shieh broth (with a concentration of 2 × 10^8^ CFU). The same bacterial concentration was used to challenge the vaccinated individuals with the pathogen. Meanwhile, sham-immunized fish underwent the same challenging procedure (14). The challenged fish were then transferred to a tank and temporarily maintained at a constant temperature of 28°C. They were observed for a week, and the cumulative mortality was recorded. The protective efficiency of *F. columnare* vaccination was expressed as the relative percent survival (RPS) of the fish. RPS was calculated using the following formula:


RPS=[1−(cumulative mortality of vaccinated group/cumulative mortality of control group)]×100%

### Serum preparation

Prior to challenging the vaccinated individuals with the pathogen, the fish were anesthetized with eugenol and blood samples were collected from the caudal vein of five fish in each group. After we anesthetized grass carp with anesthetic, we used a 2 mL syringe, inserted the needle at an angle of 45° into the tail vein of grass carp to draw blood, removed the needle of the syringe, pushed the blood in the syringe into a 1.5mL centrifuge tube, allowed it stand at room temperature for 2 hours, put in a refrigerator at 4°C overnight to precipitate more serum, centrifuged at 4000 r for 10 minutes, and then sucked the upper serum, which was stored at -20°C until needed.

### Analysis of IgMs in serum (ELISA)

The serum antibody was detected using an indirect ELISA. Preliminary tests were conducted to optimize antigen concentrations, sample dilutions, and incubation times (data not shown). The optimal conditions inferred were then used.

A 96-well flat-bottom microtiter plate was coated at 37°C for 3h with 100 µL per well of 1 µg/mL protein solution of sonicated *F. columnare* whole cell antigen in a carbonate coating buffer, (pH 9.6) (15). The plates were washed three times in PBS containing 0.05% Tween-20 (PBST) and then incubated for 4h in a blocking solution (3% BSA in PBST). The serum (1:100) was further serially diluted to 1:64000 in an incubation buffer (1% BSA in PBST) on the ELISA plate and incubated at 37°C for an hour. A rabbit anti-grass carp rIgM antibody was diluted (1:1000) with the incubation buffer. A measurement of 100 µL per well of the antibody was added for an hour. After washing the plates thrice with PBST, a goat anti-rabbit IgG antibody was diluted (1:3000) with the incubation buffer, and 100 µL of it was added per well for an hour. After washing the plates, 100 µL of TMB-ELISA substrate solution was added as well. The peroxidase reaction was stopped after 5 min with 100 µL of 3.6% HCl. The mixture was then assessed at 450 nm. An OD more than twice that of the controls was considered a positive signal.

### Statistical analysis

All statistical analyses were performed using GraphPad Prism 8. To investigate the effects and determine statistical differences in antibody titers, we performed an Analysis of Variance (ANOVA). Prior to conducting the ANOVA, we assessed the normality of the data distribution. Our analysis revealed that the data did not follow a normal distribution, even after attempting data transformations to achieve normality. Consequently, we decided to employ a non-parametric approach to analyze the data. Thus, differences in antibody titers were analyzed using a series of non-parametric tests (Kruskal-Wallis test) followed by a Dunnet’s *post hoc* test to determine which groups differ significantly from the control group at each time point.

Survival analysis was performed using the Gehan-Breslow-Wilcoxon test. The Gehan-Breslow-Wilcoxon test is a non-parametric test suitable for analyzing time-to-event data, such as survival curves. In this study, we used the Gehan-Breslow-Wilcoxon test to compare the survival curves between two groups (against the control or adjuvant group). The null hypothesis was that there was no difference in survival between the two groups, and the alternative hypothesis was that there was a significant difference in survival between the two groups. P-values less than 0.05 were considered statistically significant.

## Results

### The comparison of growth between isolate JX-01 and isolate MU-04

**Figure 1:**
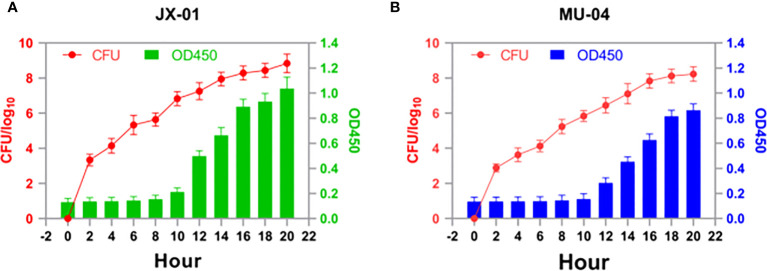
Growth profiles of **(A)** JX-01 and **(B)** MU-04. JX-01 and MU-04 were cultured at 28°C in Shieh medium. Aliquots of cell culture were taken at various time points and measured for cell density at OD450. Plate counts were carried out for each bacterial strain to calculate the number of colony-forming units per milliliter (CFU/mL).

To determine the optimal time for antigen collection, growth curves of *F. columnare* isolate JX-01 and MU-04 cultured at 28°C were established (**Figure 1**). As shown in **Figure 1**, both strains exhibited slow growth within 8 hours after inoculation and remained in a sluggish stage. Between 10 and 16 hours, the OD values of JX-01 and MU-04 reached 1.05 and 0.85, respectively. After 16 hours, the rate of bacterial reproduction tended to stabilize, reaching a plateau (CFU > 10^8^). Based on the growth curve results, *F. columnare* cultured for 20 hours was selected for vaccine preparation and pathogen challenge.

### Antigen inactivation conditions and vaccine safety

The inactivation of bacterial antigens is influenced by formaldehyde concentration, time, and temperature. Determining the most suitable conditions is crucial for preserving the surface of the bacterial antigen. **Table 3** demonstrates that bacterial culture inactivated by growing at 4°C for 24 hours in 0.025% formaldehyde resulted in colonies growing only on the plate, with no growth observed in other media. Lower temperature and shorter inactivation time better protect the bacterial surface antigens. Therefore, we concluded that exposure to 0.05% formaldehyde at 4°C for 24 hours is the optimal inactivation method. Twenty grass carp were intraperitoneally injected with 0.3 mL of inactivated bacteria and observed for a week without experiencing any abnormalities. The white oil adjuvant vaccine was mostly absorbed by the host four weeks after immunization without causing residual effects (data not shown).

**Table 3:** Conditions of formaldehyde inactivation of *F. columnare*.

Inactivation at 24 h					Inactivation at 48 h				
Concentration (%)	Temp (°C)	1	2	3	Concentration (%)	Temp (°C)	1	2	3
0.025	4	+ ^a^	+	+	0.025	4	–	–	–
	28	- ^b^	–	–		28	–	–	–
	37	–	–	–		37	–	–	–
0.05	4	–	–	–	0.05	4	–	–	–
	28	–	–	–		28	–	–	–
	37	–	–	–		37	–	–	–
0.1	4	–	–	–	0.1	4	–	–	–
	28	–	–	–		28	–	–	–
	37	–	–	–		37	–	–	–

a “+” represents colony growth on the Shieh medium.

b “-” represents no colony growth on the Shieh medium.

### Grass carp rIgM and polyclonal antibodies preparation

**Figure 2:**
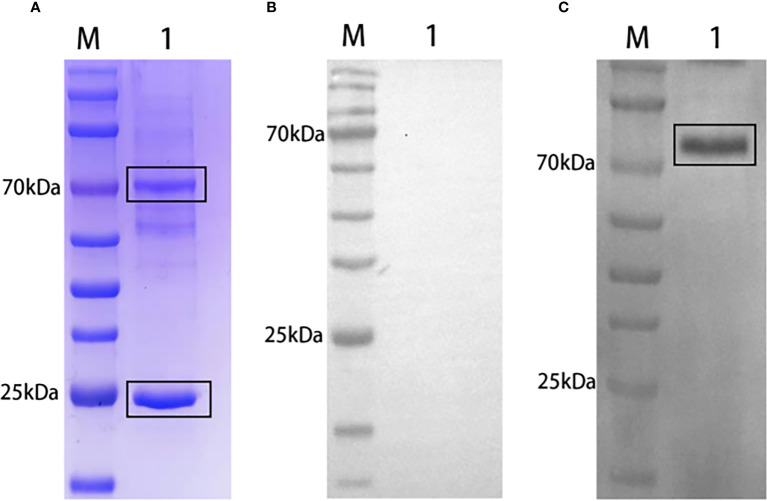
Western blot analysis of grass carp IgM. SDS-PAGE of grass carp IgM **(A)**. Lane M: Protein marker. Lane 1: Natural grass carp serum (diluted 1:20). Western blot of grass carp IgM using pre-immune rabbit serum **(B)** or rabbit anti-grass carp rIgM serum **(C)** as primary antibody. Lane M: Protein marker. Lane 1: Natural grass carp serum.

SDS-PAGE analysis of grass carp natural serum revealed a band prediction of approximately 75 kDa and 25 kDa, representing the heavy chain and light chain of grass carp IgM, respectively (**Figure 2A**). IgM from grass carp natural serum was recognized by rabbit anti-grass carp rIgM antibody (**Figure 2C**) but not by pre-immune rabbit serum (**Figure 2B**). A band of approximately 75 kDa, corresponding to the predicted size of grass carp IgM heavy chain, was detected.

### Relative percent survival of immunized fish

Two days after being challenged, infected grass carp displayed typical symptoms such as red swelling in the abdominal cavity, fin rot, loss of pigmentation on the body surface, and slow swimming, indicating the onset of mortality.

**Figure 3:**
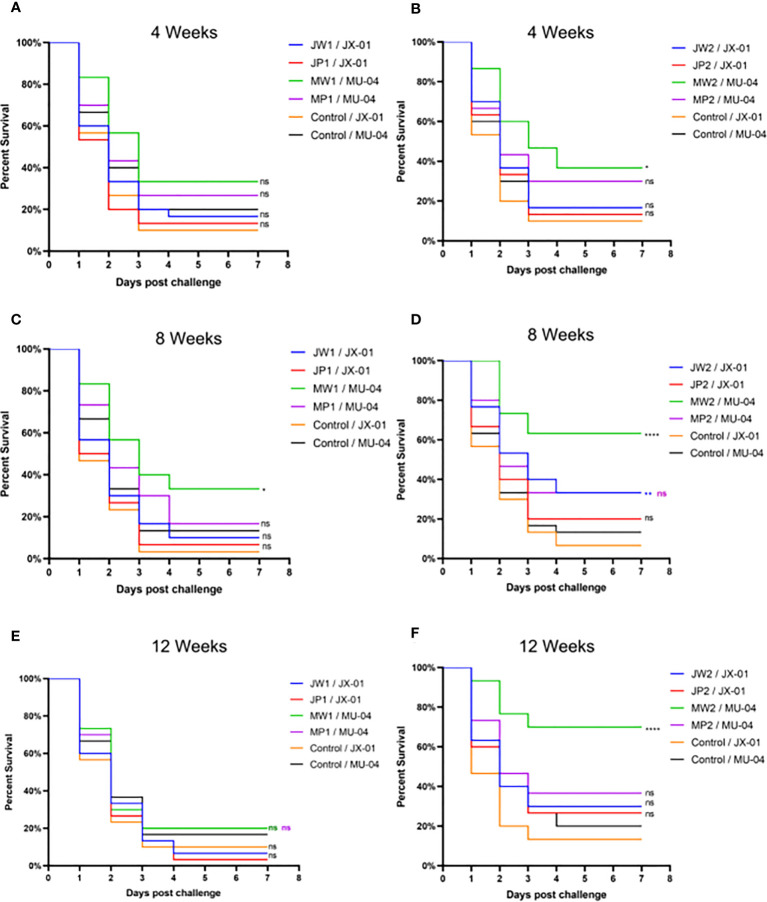
Vaccine trial schedule in Experiment 1. Percentage survival after injection challenge with 2 × 10^8^ CFU*/*mL *F. columnare* JX-01 or MU-04 strain of the non-vaccinated and different immunized groups (J/M: JX-01 or MU-04 stain; W/P: White oil adjuvant or PBS; 1/2: single or booster immunization). **(A, C, E)** Challenge results at different weeks for a single immunization group; **(B, D, F)** Challenge results at different weeks for a booster immunization group (Immunized group/Challenge stain). Statistical analysis was based on a comparison between the immune group and the control group for the same challenge strain. The symbol * indicates 0.01<p<0.05, ** indicates 0.001<p<0.01, **** indicates p<0.0001. “ns” stands for not significant (p>0.05).

**Figure 4:**
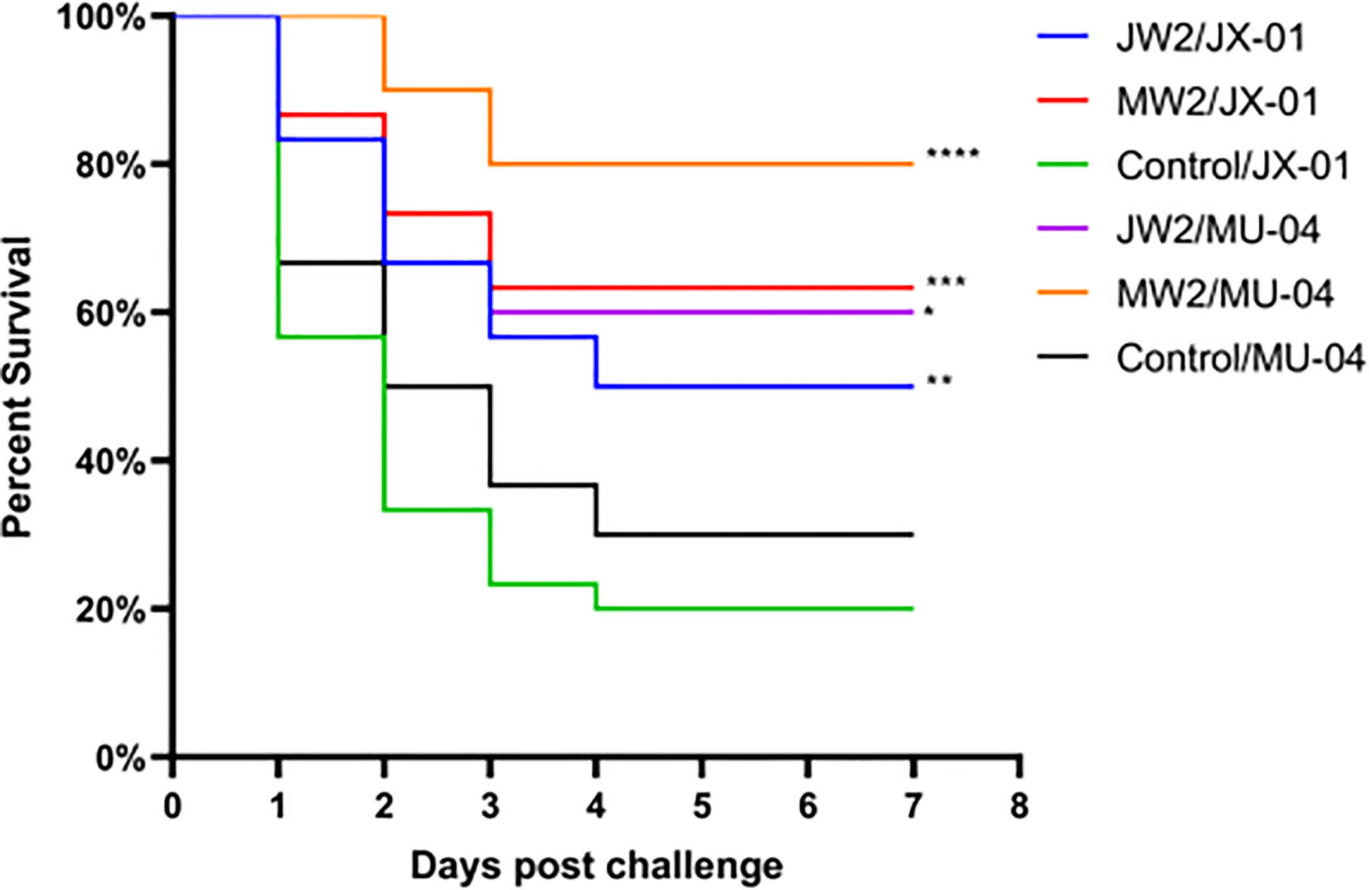
Vaccine trial schedule in Experiment 3. The cross challenge was used between immunized groups to test immune protection between cross antigens (Immunized group/Challenge stain). Two vaccine groups with better immune protection in Experiment 1 were used 8 weeks after the second immunization. Percentage survival in non-vaccinated and differently immunized groups after challenge with 2 × 10^8^ CFU*/*mL *F. columnare* JX-01 or MU-04 strains. Statistical analysis was based on a comparison between the immune group and the control group for the same challenge strain. The symbol * indicates 0.01<p<0.05, ** indicates 0.001<p<0.01, *** indicates 0.0001<p<0.001, **** indicates p<0.0001.

In Experiment 1 (**Figure 3**, **Table S1**), the protective effect of booster immunization was generally superior to that of a single immunization. The presence of white oil adjuvant yielded better results than its absence, and the duration of immunity protection was prolonged. The booster immunization was administered four weeks after the primary immunization. The RPS of the single immunization group (MW1) was highest in the eighth week (34.5%) and decreased significantly by the twelfth week (3.9%). In the booster immunization group (MW2), the RPS remained high until the eighth week (55%) and increased to 61% by the twelfth week after the primary immunization. As mentioned earlier, three *F. columnare* genomovars have been identified in grass carp, with genomovar I being the most widely distributed, followed by genomovar II and genomovar III. It was investigated whether cross-protection existed between antigens derived from different genomovar isolates. Thus, grass carp from JW2 and MW2 in Experiment 1 were used in the eighth week and cross-challenged with isolates. **Figure 4** and **Table S3** demonstrate that the MW2 group exhibited an RPS of 55% after the challenge with the JX-01 isolate, which was higher than the 32.5% RPS of the homologous immunized group. When challenged with isolate MU-04, the JW2 group also displayed 42% RPS, which was lower than the 71% RPS of the homologous immunized group. Based on these results, different genomovars can be used as antigens for full immunization of grass carp due to cross-immune protection reactions. However, genomovar II isolates provided better immune protection than genomovar I itself, offering guidance for the selection of antigen parent strains in grass carp. The results suggested that the vaccine generated using MU-04 and white oil adjuvants exhibited higher immune protection, and booster immunization with this formulation significantly improved the protection of grass carp against the *F. columnare* challenge.

**Figure 5:**
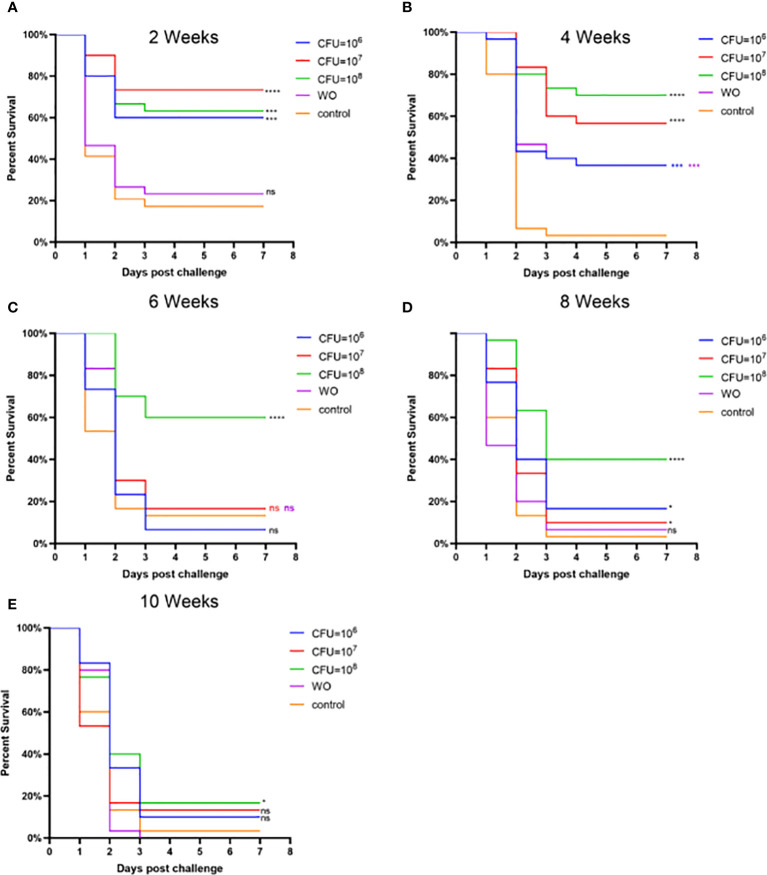
Vaccine trial schedule in Experiment 2. Isolate MU-04 is the parent strain of the vaccine. Percentage survival after challenge with 2 × 10^8^ CFU*/*mL *F. columnare* MU-04 strain was administered in different antigen doses (CFU =10^6^, 10^7^, and 10^8^) to the immunized groups at 2 **(A)**, 4 **(B)**, 6 **(C)**, 8 **(D)**, and 10 **(E)** weeks (WO: White oil group). Statistical analysis was based on a comparison between the immune group and the control group for the same challenge strain. The symbol * indicates 0.01<p<0.05, *** indicates 0.0001<p<0.001, **** indicates p<0.0001. “ns” stands for not significant (p>0.05).

Experiment 2, based on Experiment 1, explored the effects of different bacterial concentrations on the immunity of grass carp. **Figure 5** and **Table S2** present the RPS during the weeks following booster immunization. In the second week, the RPS of all three immunization groups exceeded 50%, indicating a good immune response within a short period. By the fourth week, the RPS of the MW6 and MW7 groups slightly decreased, while the RPS of the MW8 group continued to rise, reaching the highest value of 71%. From the sixth to the tenth week, the RPS of all immunized groups exhibited a continuous downward trend. Interestingly, the white oil adjuvant group also provided some immune protection, reaching 34% in the fourth week. In conclusion, a bacterial concentration of CFU = 10^8^/mL resulted in the best and relatively higher immune protection effect.

### Serum antibody titration

**Figure 6:**
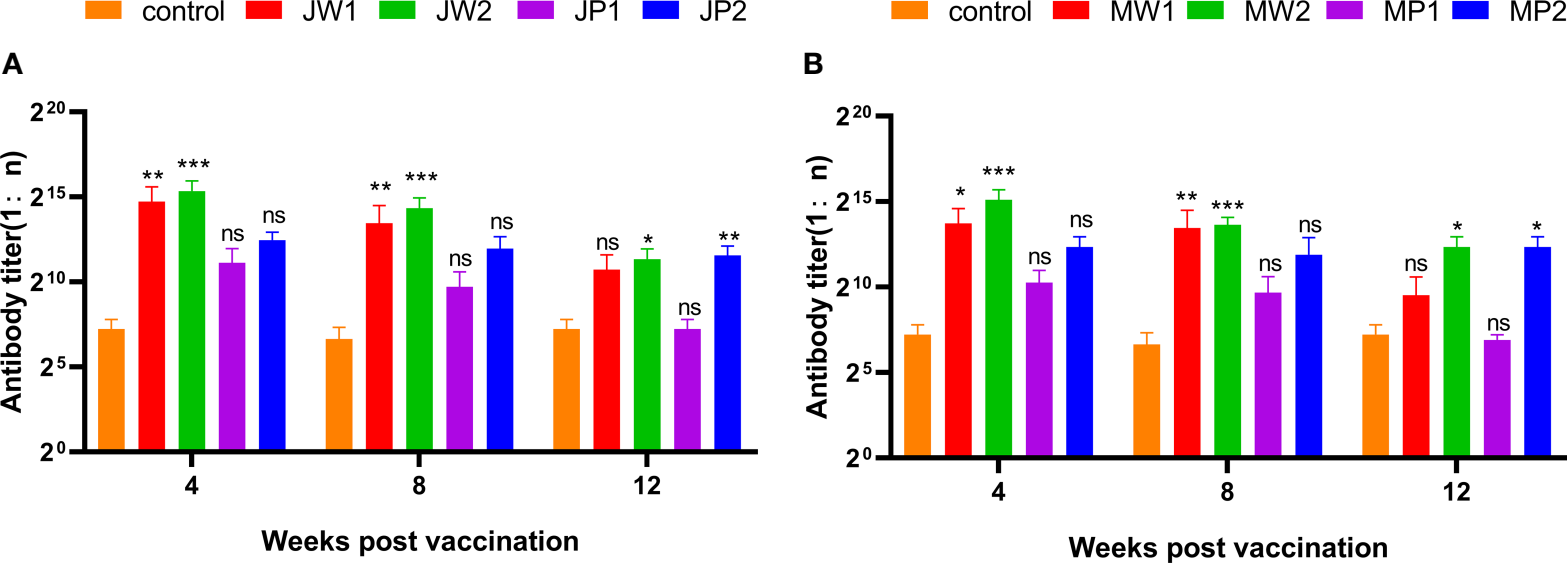
Corresponding Experiment 1: change in anti-*F. columnare* IgM antibody titer in the serum of grass carp at different weeks after the initial immunization. Data are presented as Mean ± SD. The symbol * indicates p<0.05, ** indicates 0.001<p<0.01, and *** indicates p<0.001. “ns” stands for not significant (p>0.05). **(A)** Different immunization program groups of JX-01 strain. **(B)** Different immunization program groups of MU-04 strain. (J/M: JX-01 or MU-04 stain; W/P: White oil adjuvant or PBS; 1/2: once or twice immuned).

**Figure 7:**
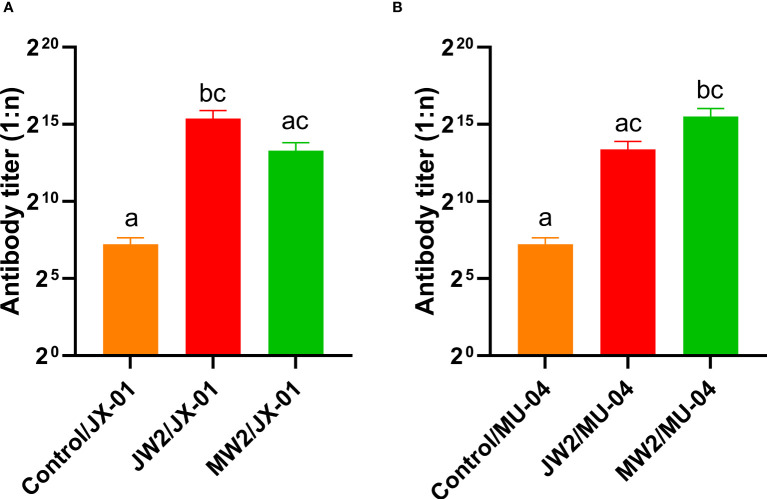
Corresponding Experiment 3: cross-antibody titer in the serum of the JW2 group and the MW2 group against JX-01 **(A)** or MU-04 antigen **(B)**. Differences in antibody titers were analyzed using a series of non-parametric tests (Kruskal-Wallis test) followed by a Dunnet’s *post hoc* test to determine which groups differ significantly from each group. Data are presented as Mean ± SEM. Different letters indicate a significant difference between the two groups (p<0.05).

**Figure 6** presents the results corresponding to Experiment 1, showing the changes in grass carp at different weeks after the initial immunization. The figure compares the antibody titers of different groups by illustrating the changes in anti-*F. columnare* IgM antibody titer in the serum. Four weeks after a single immunization, the antibody titer in the white oil adjuvant group (JW1 or MW1) was 1:20,000, while the non-adjuvant group (JP1 or MP1) had a titer of 1:2,000. The group that received booster immunization (JW2 or MW2) with the white oil adjuvant displayed antibody titers ranging from 1:48,000 to 1:62,000 in the 4th week after the initial immunization, which was 1 to 2 times higher than the primary immunization group (JW1 or MW1) with the white oil adjuvant. These findings suggest that specific IgM antibodies are generated in the serum of grass carp within 4 weeks or even earlier after the first immunization, followed by a continuous decline until the 8th and 12th week. Booster immunization with the white oil adjuvant results in a higher antibody titer. Furthermore, the results in **Figure 7** demonstrate that the cross-antibody titer in the serum of the JW2 group (against MU-04 antigen) was 1:30,000, which was equivalent to the cross-antibody titer in the serum of the MW2 group (against JX-01 antigen). The presence of cross-antibodies in both groups is not surprising, as cross-protection was observed between MU-04 and JX-01 vaccinations. This indicates the existence of cross-reactive antigens among different genomovars, providing a theoretical basis for cross-protection in the immune response.

**Figure 8:**
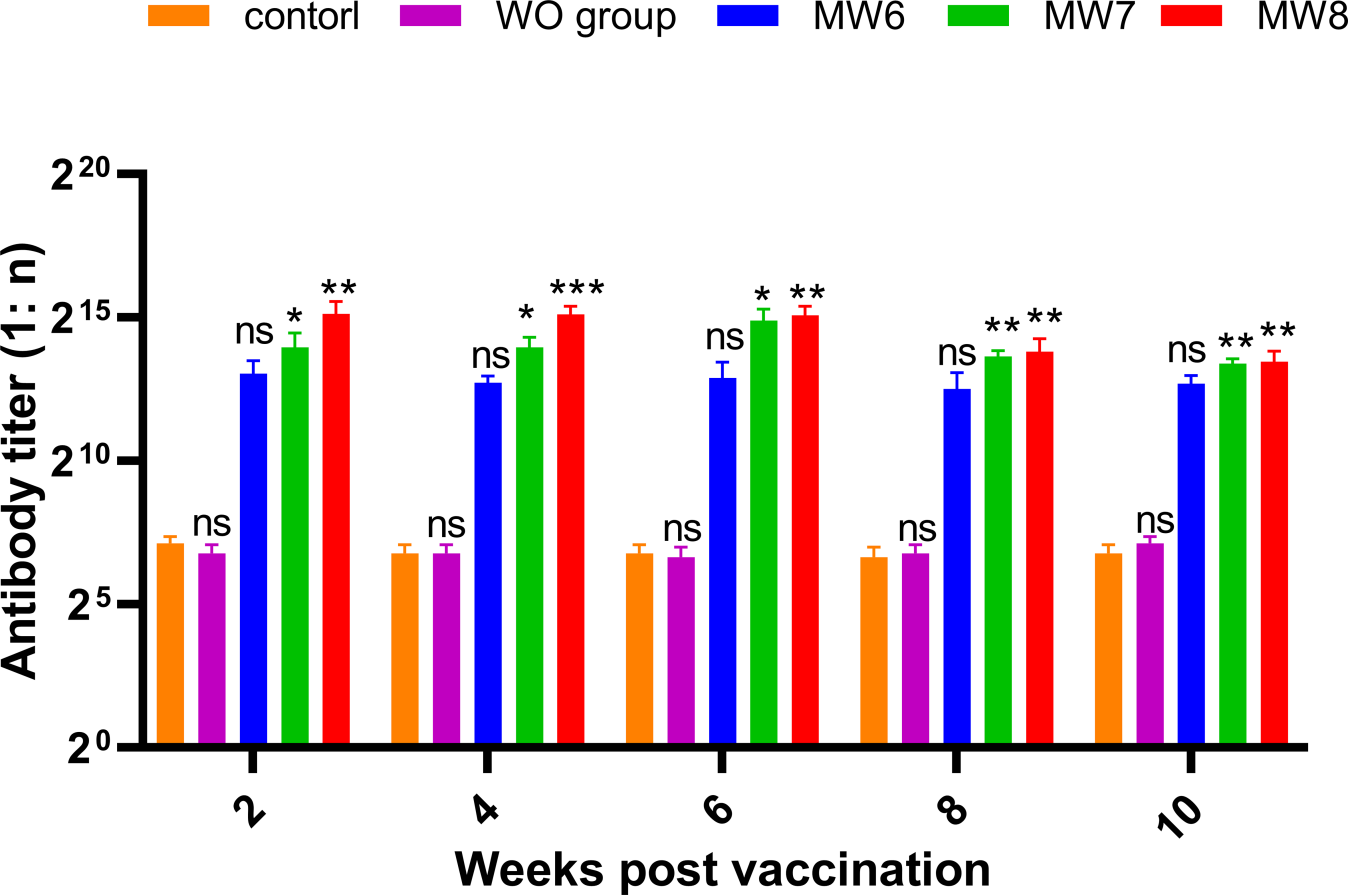
Corresponding Experiment 2: change in anti-*F. columnare* IgM antibody titer in the serum of grass carp at 2, 4, 6, 8, and 10 weeks after initial immunization, with different antigen doses (CFU = 10^6^, 10^7^, and 10^8^; WO: White oil group). Data are presented as Mean ± SD. The symbol * indicates p<0.05, ** indicates 0.001<p<0.01, and *** indicates p<0.001. “ns” stands for not significant (p>0.05).

To investigate whether different antigen doses affect the change in antibody titer in grass carp serum, Experiment 2 (**Figure 8**) was conducted. Grass carp were immunized with three concentration gradients (CFU = 10^6^, 10^7^, and 10^8^ bacteria/mL), and serum samples were collected to record antibody titers every 2 weeks after booster immunization. It was observed that antibody titers were produced as early as the 2nd week in all three immune groups, and the titers were positively correlated with the immune concentration. In the 6th week, the highest antibody titers of 1:30,000 and 1:57,000 were achieved in the MW7 and MW8 groups, respectively, while the antibody titer of the MW6 group remained at 1:10,000 from week 2 to week 6. From week 6 to week 10, the titers in all three immune groups gradually decreased. Based on these data, higher immune doses can induce the production of serum antibodies in grass carp post-vaccination.

## Discussion

A wide range of fish species can be infected with *F. columnare*, resulting in significant losses in aquaculture. In China, the main epidemic season for *F. columnare* occurs from June to August. Vaccination of grass carp at an appropriate time and using a suitable method can help resist pathogen invasion and reduce disease-related losses (16). Various factors, such as temperature, fish age, nutritional status, vaccine type, and physicochemical characteristics of the vaccine solution, can influence antigen uptake and immune efficacy (17). In this study, we compared the effects of different application procedures of the inactivated *F. columnare* vaccine.

In China, three genomovars of *F. columnare* have been isolated from grass carp, with most isolates belonging to genomovar I and only a few to genomovar II and III (9). As grass carp are susceptible to different genomovars of *F. columnare* in the natural environment, strain selection is crucial in vaccine development. In this study, we selected isolates JX-01 (genomovar I) and MU-04 (genomovar II) as candidate strains for immunizing grass carp. Our results showed that the MW2 group exhibited 55% relative percent survival (RPS) after being challenged with isolate JX-01, which was higher than the RPS of 32.5% in the homologous immunized groups. Therefore, we chose isolate MU-04 as the vaccine strain for Experiment 2. Our data indicated that common antigens are likely shared among genomovars although genomovar II provided higher protection than genomovar I. Similar findings were reported in a previous study, where a genomovar II mutant and FCRR strain provided protection against columnaris caused by genomovar I (4).

Freund’s complete adjuvant has been shown to induce a significant humoral response to *F. columnare* in tilapia (18); however, it is costly and has undesired side effects. Therefore, we used low-cost white oil as an adjuvant. Previous studies have reported that vaccines emulsified in white oil adjuvant have long-lasting effects on RPS and serum antibody titers (11). Consistent with these findings, our study demonstrated that the MW2 group at 8 weeks in Experiment 1 and the MW8 group at 4 weeks in Experiment 2 exhibited a greater protective effect than the other groups during the same period. The addition of adjuvants resulted in 55-71% RPS post-challenge, indicating the effectiveness of this procedure. It is worth noting that even the white oil adjuvant group in Experiment 2 played a role in immune protection, providing 34% RPS post-challenge at week 4. Since white oil does not contain *F. columnare* antigens, it does not directly stimulate B cells to produce specific antibodies. However, the activation of non-specific immunity by white oil itself may contribute to immune protection against *F. columnare* (19). Nevertheless, our findings confirmed that the addition of white oil adjuvant can enhance the effectiveness of the *F. columnare* vaccine.

To determine the optimal vaccine dose, we immunized grass carp with different antigen concentrations. Previous research has shown that specific immunity increases with all three doses (10^5^, 10^7^, and 10^10^ CFU/mL) compared to the control, with a dose-dependent effect (20). In our study, a dose of 10^8^ CFU/mL resulted in the best immune protection. It is not surprising that a higher antigen concentration leads to greater antigen uptake by the host’s immune cells, thereby enhancing their ability to present antigens. When combined with the encapsulating effect of the white oil adjuvant, the antigens act as a reservoir that continuously stimulates the immune system. We selected a concentration of 10^8^ bacteria/mL as the highest inoculation dose, which corresponds to the growth limit value identified from the plateau of the growth curve for *F. columnare*. Earlier studies have also shown that the antibody response in carp to intraperitoneal doses of 10^10^ and 10^13^ CFU/mL (and above) does not significantly differ (21). Moreover, higher doses need to be concentrated through re-suspension, which reduces bacterial secretion proteins and increases production costs. Overall, we suggest that a concentration of 10^8^ bacteria/mL is optimal for the development of an *F. columnare* vaccine.

As fish are poikilotherms, their immune response to invading pathogens is strongly influenced by environmental temperature (17). In aquaculture, water temperature changes according to the local climate. To confirm the effectiveness of the vaccine, we raised grass carp in outdoor ponds where the water temperature positively influenced immunity. In Experiment 1, the water temperature increased from 24 to 29°C from April to July, and the maximum RPS was observed during weeks 8 to 12. In Experiment 2, the water temperature decreased from 30 to 23°C from August to October, and the maximum RPS was observed at 4 weeks. Water temperature is considered an important factor controlling the efficacy of vaccination (22). It affects the teleost immune system, altering susceptibility to pathogenic organisms and the induction of immunological protection after vaccination (23). Therefore, vaccination at a temperature of approximately 26-30°C is recommended.

In Experiment 1, the white oil adjuvant booster immunity group showed a relative immune protection rate at week 8, but the highest antibody titer in the serum of grass carp in this group appeared at week 4. Though this could be interpreted as an indication that the antibody did not play the primary role in imparting disease resistance, this is not necessarily the case. In fact, under two immune stimulations, the body can produce higher levels of serum antibody titers within a few weeks. Once the antigen has been absorbed by the body, it is gradually consumed, resulting in a decrease in antibody levels due to its half-life. In the absence of antigens to sustain antibody stimulation, serum antibody levels gradually decrease, and more antibodies are converted into memory effector B cells, which can rapidly produce a large number of antibodies in response to subsequent pathogen invasions. Sufficient humoral immunity can be induced when antibodies reach a certain threshold in the serum, and excessive antibodies are not beneficial in resisting pathogens. Additionally, the role of nonspecific immunity should not be overlooked. Further research is needed to investigate the underlying mechanisms of action of the vaccines developed in this study.

While high antibody titers generally indicate high immune protection, specific serum antibody titers do not always guarantee higher protection (24). Furthermore, the protection provided by vaccines shows a moderate linear correlation with antibodies in mucus but a low linear correlation with antibodies in serum (25, 26). In our study, we observed the highest immune protection in the white oil adjuvant group at week 8, but the highest antibody titer was detected in the serum of the same group at week 4, which declined by week 8. Excessive antibodies in the serum may not contribute to the defense against pathogen invasion. Moreover, maintaining high levels of specific antibodies in the absence of a pathogen can burden the host. Therefore, in this situation, the innate immune system may play an auxiliary role in eliminating *F. columnare*. Further research is needed to investigate the contribution of innate immune components, such as macrophages or other complement factors, to immune protection against *F. columnare* following vaccination.

In conclusion, *F. columnare* is widely present in the aquatic environment and often causes outbreaks in China’s aquaculture industries during the spring and summer seasons. Vaccination provides effective protection within a specific period. Vaccination timing should coincide with the epidemic season to reduce losses in grass carp farming. It is crucial to determine the appropriate time points and immune protection periods during the development of *F. columnare* vaccines. Based on the RPS and antibody titer results, the booster dose of the MU-04 strain with white oil adjuvant elicited the highest immune response in vaccinated grass carp, resulting in the highest efficacy of immune protection. This formulation could be considered as a reference for the injection of inactivated *F. columnare* vaccine in practical applications for grass carp.

## Data availability statement

The original contributions presented in the study are included in the article/**Supplementary Material**. Further inquiries can be directed to the corresponding authors.

## Ethics statement

The animal study was reviewed and approved by the ethical guidelines of the Animal Research Ethics Committee of South China Agricultural University, China. The care and treatment of animals were performed in accordance with the ethical guidelines of the committee (approval No. 2022G009). Written informed consent was obtained from the owners for the participation of their animals in this study.

## Author contributions

WG, XD, ZM, and YL conceived and designed the experiments and provided the original idea of the study, and also contributed reagents, materials, and tools. WG, RH, WX, and ZL performed the experiments. WG wrote the first draft of the manuscript and was involved in all aspects of the study. All authors contributed to the article and approved the submitted version.
