# Supplementary material for: The protective effect of inactivated Flavobacterium columnare vaccine in grass carp (Ctenopharyngodon idellus)
Source: Front Immunol. 2023 Jul 13;14:1162975. doi: 10.3389/fimmu.2023.1162975 (PMC10381957; doi:10.3389/fimmu.2023.1162975)

**S Table 1**

Relative percent survival of fish immunized in Experiment 1

**S Table 2**

**Experiment 2: Relative percent survival of immunization with different bacterial concentrations**

| RSP (%) | | | | | |
| --- | --- | --- | --- | --- | --- |
|  | Time after vaccination / w | | | | |
| CFU | 2 week | 4 week | 6 week | 8 week | 10 week |
| 1.7 × 10^6^ | 51 | 38 | 12 | 13.5 | 4.3 |
| 1.7 × 10^7^ | 68 | 57 | 23 | 6.2 | 10.6 |
| 1.7 × 10^8^ | 56 | 71 | 64 | 37.5 | 13.5 |
| White oil | 13 | 34 | 23.8 | 3.1 | -4.5 |

**S Table 3**

Experiment 3: Relative percent survival of antigenic cross challenge

| RPS (%) | | |
| --- | --- | --- |
|  | Challenge stain | |
| Group | JX-01 | MU-04 |
| JW2 | 32 | 42 |
| MW2 | 55 | 71 |

**S Figure 1**

Changes in water temperature under the local climate in this study.


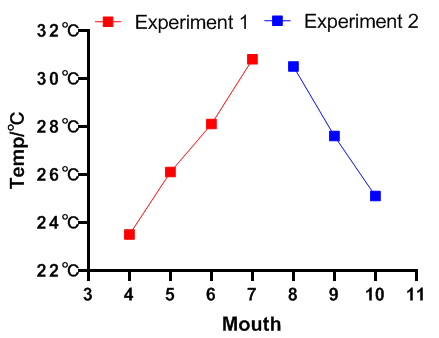

Supplement: Supplementary file 1 [file DataSheet_1.doc]
