# Supplementary material for: The protective effect of inactivated Flavobacterium columnare vaccine in grass carp (Ctenopharyngodon idellus)
Source: Front Immunol. 2023 Jul 13;14:1162975. doi: 10.3389/fimmu.2023.1162975 (PMC10381957; doi:10.3389/fimmu.2023.1162975)
Supplement: Supplementary file 2 [file DataSheet_2.doc]

**Supplement materials**

**S** **FIGURE 1** Comparison of virulence of different genomovar strains


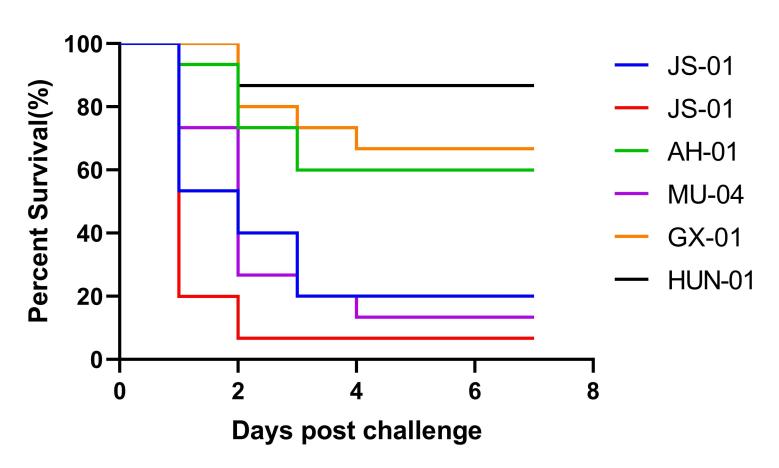


**S FIGURE 2** The pET32a-ΔTRX-grass carp-IgM plasmid


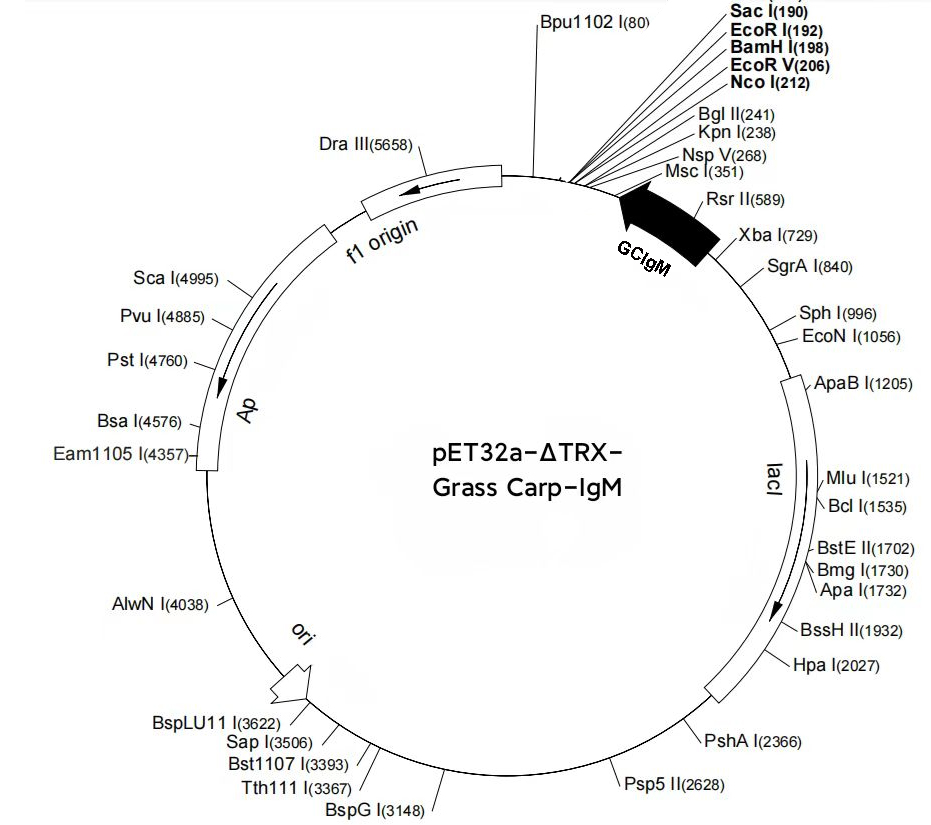


**DNA sequence**

ATGCCAGATCAGCCTGCAACTGTCTACTTAACAGTACCTACACAAAAGGATTTAGAAAATGGAACAGCAACCTTCTTGTGTTTAGCCCAGCAGTTTTCACCTAAAAAATATTCGTTTAAGTGGTTTAAGGATGGTCATCAGGTGGCAAATACAATAAACACCTATGACACAAGTGAGAAGAATGGCTCAGTAACCTTATATAGCGCCACAAGCAGTTTGCAAATCAGTGCCGAAGAATGGAAGACAGCCGCCAAAATCAAGTGCGAGTTTGAGCACAAGACGGGAAAAGAAGTCAGAGAGGCCGCATATACAGATAATAATCATGATGACTGCACCAATGTTGCTGCTGTTATAGTCCCCCCGTCCCTTGAAGACATGCTGAAAAATAGAGAAGGAACGCTGACGTGCAAAGCTTCAGGAGCAAATCCAGGATTCACCAAAATAGAGATAAAAGCAAATAATTTTGTCATCGCTGAGGCATCGGAGGCACATTTCAAGAACAAAATAAAGGTGGAGCTTGAAGCCCCTATAGGCTACGAAGAATGGAGCAACGGCACAGTATTTACGTGCACAGTTGAACACACAAAACTACCACAGTAA
